# Correlation of Ventricular Arrhythmias With Peak and Trough Levels of Oral Digoxin: A Prospective Ambulatory Electrocardiographic Study

**DOI:** 10.7759/cureus.113522

**Published:** 2026-07-28

**Authors:** Jyothi Vijay, Ajay Bahl, Neelam Dahiya, Himanshu Gupta

**Affiliations:** 1 Department of Cardiology, Sree Chitra Tirunal Institute for Medical Sciences and Technology, Thiruvananthapuram, IND; 2 Department of Cardiology, Postgraduate Institute of Medical Education and Research, Chandigarh, IND

**Keywords:** arrhythmias, atrial fibrillation, cardiomyopathy, digoxin, heart failure, ventricular ectopic

## Abstract

Introduction: Digoxin is an affordable medication with a narrow therapeutic window and is widely used globally, particularly in resource-constrained areas. Once-daily oral digoxin produces intraday fluctuations in plasma concentration, as expected from its established pharmacokinetic profile. Whether these fluctuations translate into a time-dependent increase in arrhythmia frequency has not previously been examined in a prospective setting. Given its narrow therapeutic index, there is always concern about digoxin's proarrhythmic potential. We postulated that ectopic arrhythmia density would be greatest during the period of highest drug exposure and least during the pre-dose nadir.

Methods: This prospective observational study included 50 patients with indications for digoxin therapy, including heart failure with reduced ejection fraction (HFrEF) or rheumatic heart disease (RHD), who underwent 24-hour Holter monitoring. Digoxin 0.25 mg was administered four hours after the Holter was attached, under direct physician supervision. The 24-hour recording was segmented into six four-hour intervals relative to drug intake. The period before ingestion was considered the trough phase. The two- to six-hour post-ingestion interval was identified as the pharmacokinetic peak phase, based on existing digoxin pharmacokinetics data. The main goal was to compare ventricular ectopic beat (VEB) burden across these intervals using the Friedman test with Nemenyi post-hoc correction.

Results: The cohort had a mean age of 50.6±13.4 years; 58% were male. Underlying diagnoses were non-ischaemic dilated cardiomyopathy (n=28, 56%), ischaemic cardiomyopathy (n=7, 14%), and RHD (n=15, 30%). Atrial fibrillation was present in 48%. The median VEB count during the designated peak phase (hours 2-6) did not differ significantly from that in any other four-hour interval (Friedman χ²=3.3, p=0.767). Pairwise comparisons of all intervals with the peak phase showed no significant difference (all p>0.05, Nemenyi correction). No sustained ventricular tachycardia was recorded in any participant.

Conclusions: In patients on stable oral digoxin therapy, ventricular ectopic burden does not correlate with the drug's peak or trough pharmacokinetic levels. These findings suggest that the proarrhythmic risk of digoxin at therapeutic doses is less likely to be driven by intra-day fluctuations in drug levels. Larger controlled studies with concurrent serum level monitoring are needed to confirm this observation.

## Introduction

Digoxin occupied a well-defined place in contemporary cardiovascular practise as an adjunct therapy in heart failure with reduced ejection fraction (HFrEF) and for rate control in atrial fibrillation in symptomatic rheumatic heart disease [1,2]. Digoxin provides a positive inotropic effect in heart failure. In atrial fibrillation, digoxin's rate-controlling properties are beneficial [2,3].

The landmark DIG trial established that digoxin therapy reduces the composite of death or hospitalisation from worsening heart failure without improving overall mortality but noted a non-significant excess of arrhythmic fatalities in the active treatment arm. This has attracted considerable attention and has influenced prescribing behaviour ever since due to concerns of proarrhythmic effects. Subsequent observational data have reinforced this concern [4,5].

An increased risk of ventricular tachycardia and ventricular fibrillation was observed with digoxin use in the MADIT-CRT registry [4]. Data from the TREAT-AF registry demonstrated a graded relationship between digoxin exposure and all-cause mortality in patients with incident AF [5,6]. The heightened risk correlates with higher digoxin levels. A large analysis of the ARISTOTLE trial confirmed that digoxin levels over 1.2 ng/mL were associated with a 56% higher mortality risk [7].

We hypothesised that arrhythmias may rise at peak digoxin levels and fall at trough levels. Once-daily oral administration inevitably produces a transient post-absorption concentration peak before redistribution and elimination restores the trough level. If arrhythmias cluster around the pharmacokinetic peak, this will have direct clinical implications, favouring modified-release formulations, divided dosing, or timing adjustments. Conversely, if arrhythmia burden is independent of intra-day concentration variation, this would suggest that substrate factors, rather than acute concentration changes, may be causing the proarrhythmic risk. To address this unresolved question, we conducted a prospective ambulatory electrocardiographic study in which we observed the ectopic arrhythmia frequency across six consecutive four-hour segments of a single dosing cycle and tested whether the pharmacokinetic peak window carried excess arrhythmia burden relative to the pre-dose trough.

Preliminary findings from this work were presented as an abstract poster at the Asia Pacific Heart Rhythm Society (APHRS) 2024 conference, held in Sydney, Australia.

## Materials and methods

This was a prospective observational study involving patients undergoing digoxin therapy at the Postgraduate Institute of Medical Education and Research, a tertiary care centre in northern India. The study received approval from the Institutional Review Board (IRB) of the Postgraduate Institute of Medical Education and Research (approval number: INT/IEC/2018/002067). Written informed consent was obtained from all participants after the study procedures were explained. The study was conducted in accordance with the principles of the Declaration of Helsinki, from December 2018 to January 2020.

Patients with heart failure and severe LV systolic dysfunction (LVEF less than 35%) or rheumatic heart disease (RHD), irrespective of baseline LVEF, were included. All patients were already on oral digoxin therapy. Patients aged ≥18 years who were already receiving oral digoxin 0.25 mg once daily for a minimum of five days (to ensure pharmacokinetic steady-state) for an established clinical indication were eligible. Hence, it was assumed that patients had achieved steady-state concentrations [8]. The last dose was taken 24 hours before, as prescribed.

Individuals were excluded if they had active thyroid dysfunction, sinoatrial or atrioventricular nodal disease, a recognised inherited arrhythmia syndrome, premature ventricular complexes at baseline, current pregnancy, a febrile intercurrent illness, severely impaired renal function (estimated glomerular filtration rate below 30 mL/min/1.73 m²), acute cerebrovascular injury, hyperkalaemia, hypokalemia, or a myocardial infarction within the preceding three months. Any participant who smoked or ingested alcohol during the recording period was excluded from the final analysis.

A continuous 24-hour ambulatory ECG was recorded using a Holter system (Mortara Instrument Inc., Milwaukee, USA). The monitor attachment preceded the study dose by exactly four hours, establishing an initial pre-dose observation window and allowing time to adjust to the Holter machine (and to reduce anxiety). At hour zero, defined as the moment of drug ingestion, a single tablet of digoxin 0.25 mg was administered under direct medical supervision. All concomitant medications were maintained without alteration throughout the recording period. Each recording was subjected to automated beat classification followed by systematic manual verification by an experienced technician and verified by a cardiologist.

Due to resource limitations, digoxin levels were not measured. Instead, the timing of the peak effect was estimated based on established digoxin pharmacokinetics reported in the literature [9]. Peak serum digoxin levels are achieved around 30 minutes after drug ingestion in a fasting state and 90 minutes after in the postprandial state in healthy subjects [10]. Peak serum levels are achieved in 1.6 hours in patients with advanced right-heart failure [11,12]. The onset of clinical effect is reported within 1-3 hours and peaks at 2-6 hours post-ingestion [9]. Digoxin has a half-life of 35 to 48 hours [9,12].

The 24-hour recording was divided into six non-overlapping four-hour segments referenced to hour zero: the pre-dose segment (−4 to 0 h, designated the trough reference) and five post-dose segments (0 to +4 h; +4 to +8 h; +8 to +12 h; +12 to +16 h; +16 to +20 h). An additional overlapping analysis window spanning +2 to +6 h was separately examined as the pre-specified pharmacokinetic peak interval. This designation was based on the pharmacokinetics of digoxin [9]. The four-hour interval was selected based on practical clinical and methodological considerations.

Since digoxin's peak effect was anticipated to occur 2-6 hours after taking it, this period was analysed separately and compared with the other four-hour intervals. A comparison between subgroups of patients with high (10-100 beats per hour) and very high (>100 beats per hour) ectopic burdens was also conducted.

Sample size and power

No comparable prospective dataset was available in the literature at the time of study design development to permit a conventional power calculation. To the best of our knowledge, no prior work has specifically examined the correlation between intra-dose digoxin concentration fluctuations and intra-day ventricular ectopic variation, which is the focus of the current study.

Accordingly, this prospective investigation was designed as an exploratory, hypothesis-generating study to address this gap in the literature. The pragmatic sample size of 50 patients was determined based on recruitment feasibility at our institution, consistent with methodological guidance for hypothesis-generating research in understudied areas [13]. A formal pre-specified sample size calculation was not performed due to the absence of prior data on intra-day arrhythmia variation in digoxin-treated patients [13].

Our findings should be interpreted as hypothesis-generating. These data may be useful for sample size estimation for future confirmatory trials.

Statistical analysis

Data were analysed using IBM SPSS Statistics for Windows, Version 26 (Released 2019; IBM Corp., Armonk, New York). Data were collected using a pre-set proforma. Continuous, normally distributed variables are expressed as mean ± SD; non-normally distributed variables as median (interquartile range). Categorical variables are presented as frequencies and percentages; between-group comparisons used the chi-squared test with Yates’ correction or Fisher’s exact test, as appropriate. A repeated-measures comparison of ectopic burden across the six intervals used the Friedman test for non-normally distributed data, with post-hoc pairwise comparisons using the Nemenyi correction. Both median and mean have been added for clinically meaningful information. It serves distinct purposes: the median shows central tendency, while the mean shows the magnitude of heterogeneity. A two-sided p-value <0.05 was considered statistically significant.

## Results

Cohort characteristics

All 50 enrolled participants completed the protocol and were included in the final analysis. The group was predominantly male (n=29, 58%), with a mean age of 50.6±13.4 years. The largest diagnostic subgroup comprised non-ischaemic dilated cardiomyopathy (NIDCM; n=28, 56%), followed by RHD (n=15, 30%) and ischaemic cardiomyopathy (ICM; n=7, 14%). Left ventricular function was severely impaired in the majority of cardiomyopathy patients: 26 individuals (52%) had LVEF at or below 30%, and 10 (20%) had LVEF at or below 20%. In the NIDCM subgroup, the mean LVEF was 23.4±4.3%; in the ICM subgroup, it was 23.0±5.2%. Twenty-four participants (48%) carried a concurrent diagnosis of AF. Detailed baseline characteristics, including between-group comparisons across the three aetiological subgroups, are shown in Table 1. Inter-group differences were confined to clinically expected variables, AF prevalence (p=0.001), prior myocardial infarction (p=0.007), and smoking history (p<0.001).

**Table 1:** Baseline and demographic characteristics of the study cohort by aetiological subgroup. Different aetiological subgroups of the study population are tabulated. The data are presented as n, %, and mean±SD. *Fisher’s exact test was used. A p-value <0.05 was considered statistically significant. NIDCM: non-ischaemic dilated cardiomyopathy; ICM: ischaemic cardiomyopathy; RHD: rheumatic heart disease; LVEF: left ventricular ejection fraction; SD: standard deviation.

Parameter	NIDCM (n=28)	ICM (n=7)	RHD (n=15)	p-value
Age in years (mean±SD)	50.9±14.3	57.1±10.3	47.1±12.4	0.121
Male sex (n, %)	17 (60.7%)	6 (85.7%)	6 (40.0%)	0.197
Hypertension (n, %)	2 (7.1%)	2 (28.6%)	0 (0.0%)	0.070
Diabetes mellitus (n, %)	1 (3.6%)	1 (14.3%)	0 (0.0%)	0.349
Prior myocardial infarction (n, %)	2 (7.1%)	4 (57.1%)	1 (6.7%)	0.007*
Smoking (n, %)	2 (7.1%)	5 (71.4%)	2 (13.3%)	<0.001*
Atrial fibrillation (n, %)	9 (32.1%)	2 (28.6%)	13 (86.7%)	0.001*
Mean LVEF (%)	23.4±4.3	23.0±5.2	Normal	-

Heart rate profile on ambulatory recording

Across the 24-hour recordings, the group mean heart rate was 78.5±10.1 bpm (range: 60-101). The minimum recorded rate was 59.4±13.7 bpm (range: 30-117 bpm), and the maximum was 117.7±23.3 bpm (range: 64-175 bpm). No participant had a recorded episode of sustained ventricular tachycardia during the monitoring period.

Ventricular ectopic beat (VEB) burden

Thirty-nine patients (78%) had a VEB burden of <10/hour (group 1), eight (16%) had a burden of 10-100/hour (group 2), and three (6%) had a burden of >100/hour (group 3). The mean total VEB count over 24 hours was 504 beats. VEB counts across the six four-hour intervals are presented in Table 2.

**Table 2:** Ventricular ectopic beat frequency across consecutive four-hour recording segments (n=50). *Pre-specified pharmacokinetic peak window. χ² and p-value refer to the Friedman test, which was used across all six non-overlapping intervals. A p-value <0.05 was considered statistically significant. h: hours

Recording segment	Mean (SD)	Median (IQR)	Range	χ²	p-value
−4 to 0 h — trough	54.5 (136.9)	6.5 (18.0)	0–641	3.3	0.767
0 to +4 h	65.7 (195.1)	10.0 (19.8)	0–1102		
+2 to +6 h — peak*	87.0 (282.0)	5.0 (38.0)	0–1845		
+4 to +8 h	94.8 (342.5)	5.0 (29.3)	0–2259		
+8 to +12 h	45.1 (92.3)	5.5 (33.3)	0–483		
+12 to +16 h	89.0 (238.5)	7.5 (27.0)	0–1293		
+16 to +20 h	88.9 (222.1)	9.0 (30.3)	0–978		

Median VEB counts in the peak phase (hours +2 to +6: median 10.0, IQR 19.75) did not differ significantly from the trough phase (hours −4 to 0: median 6.5, IQR 18.0) or from any other interval (Friedman χ²=3.3, p=0.767). Pairwise comparisons of all intervals against the peak phase showed no significant differences after Nemenyi correction (Table 3).

**Table 3:** Post-hoc pairwise comparisons of VEB count between each recording segment and the pharmacokinetic peak window (+2 to +6 h). ^†^Nemenyi post-hoc correction was used for comparison. A p-value <0.05 was considered statistically significant. h: hours; VEB: ventricular ectopic beat

Segment versus peak window	Mean difference (SD)	Median difference (IQR)	Difference range	p-value^†^
Trough (−4 to 0 h)	−11.1 (83.5)	0.0 (5.8)	−494 to +179	1.000
0 to +4 h	+21.4 (171.4)	0.0 (13.5)	−670 to +743	1.000
+4 to +8 h	+29.2 (227.1)	−1.0 (11.8)	−694 to +1157	0.969
+8 to +12 h	−20.6 (179.6)	0.0 (16.0)	−974 to +412	1.000
+12 to +16 h	+23.4 (122.1)	0.0 (13.5)	−261 to +726	1.000
+16 to +20 h	+23.2 (134.9)	0.0 (6.8)	−224 to +818	0.994

**Figure 1:**
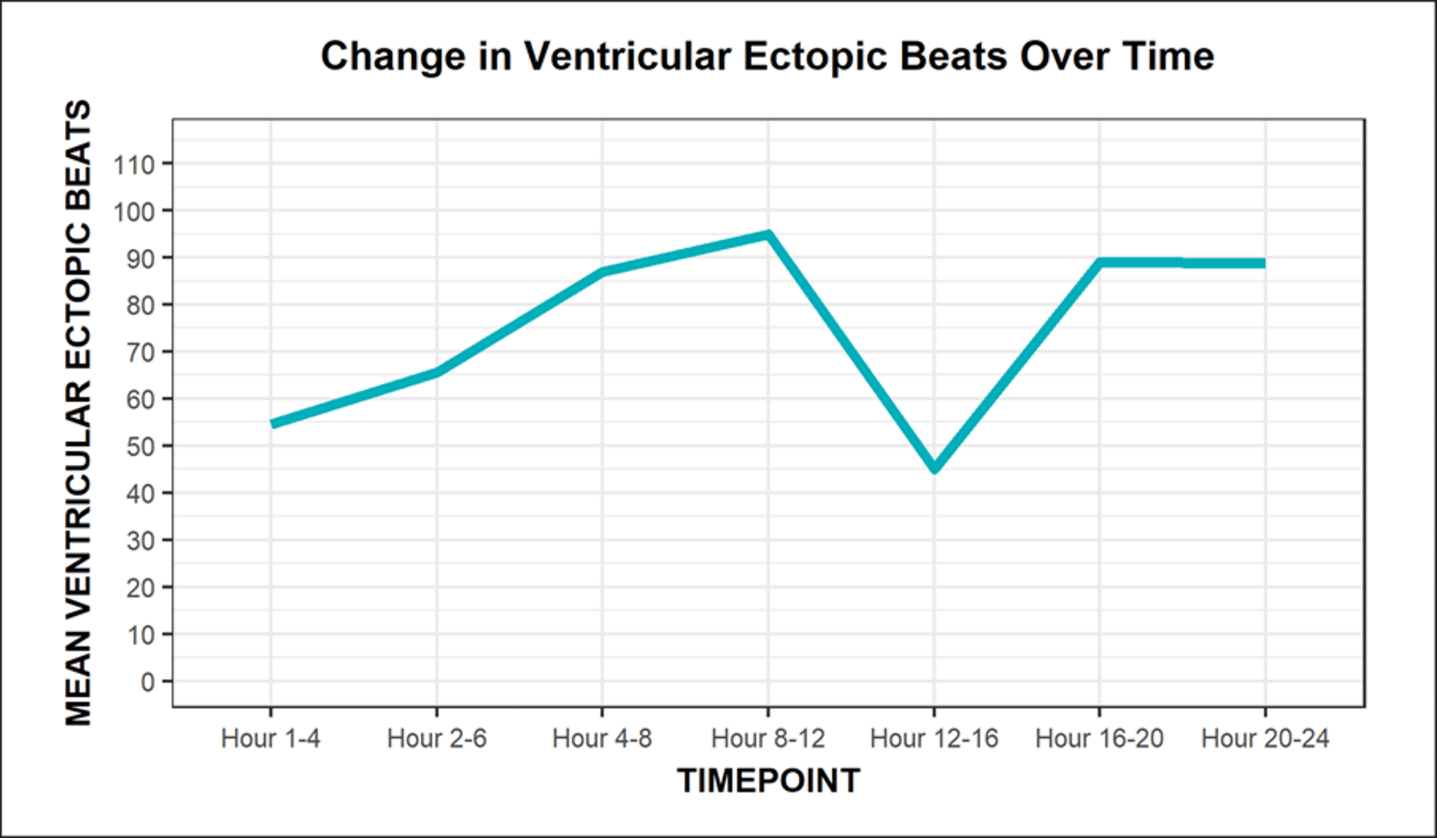
Line diagram depicting the change in ventricular ectopic burden over time. Various time segments are shown.

Subgroup analyses in patients with high (group 2) and very high (group 3) VEB burden likewise revealed no significant temporal clustering around the peak phase (Figure 1).

**Figure 2:**
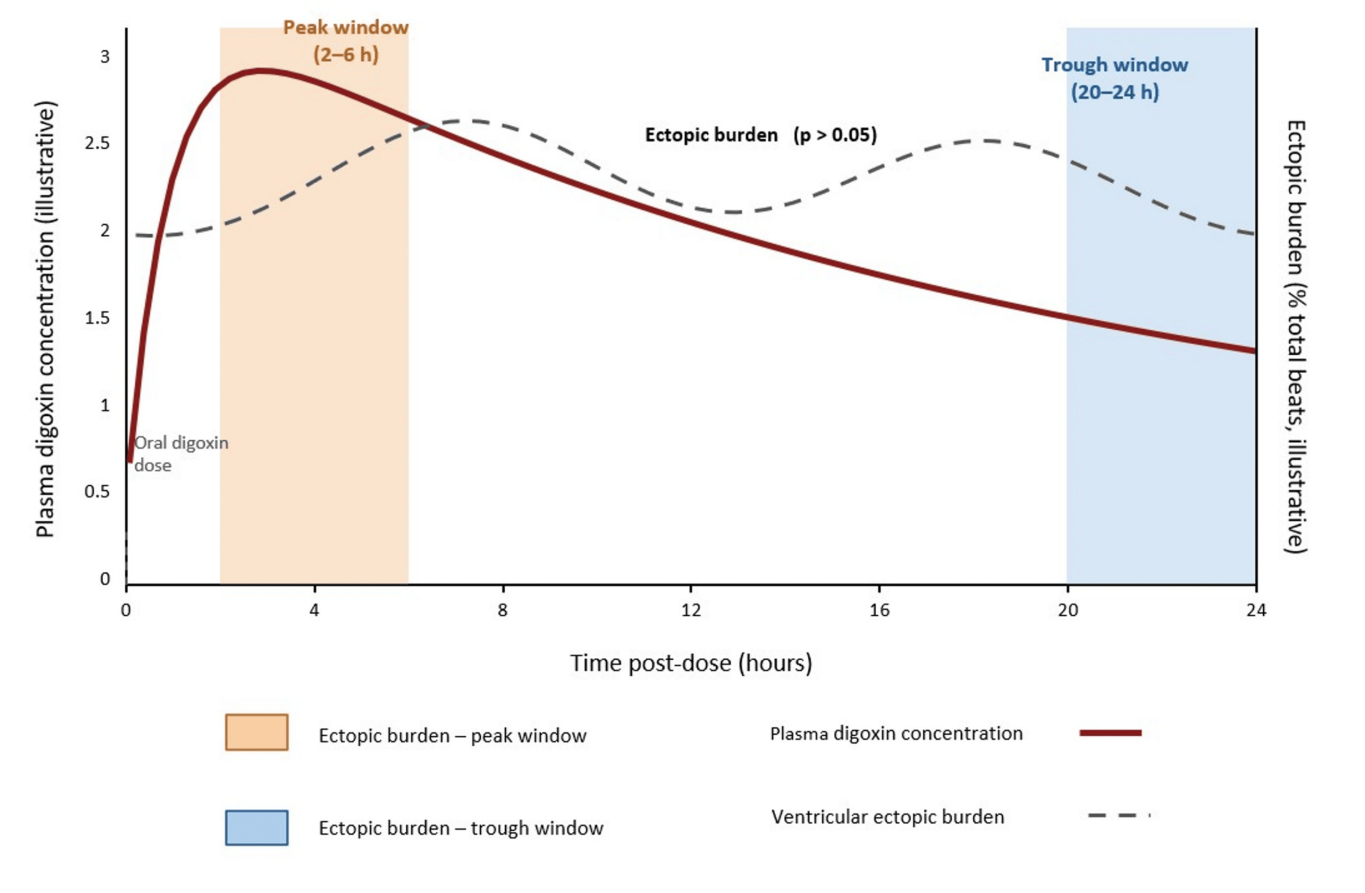
Schematic diagram illustrating the pharmacokinetics of digoxin and a 24-hour Holter time segmented four hourly. Peak and trough plasma windows show comparable ectopic burden (p>0.05). The peak and trough windows are highlighted in different colours, with a brown line indicating variation in digoxin levels according to the pharmacokinetics of digoxin as per known literature and a dashed line indicating the ventricular ectopic burden (schematic drawing not to scale).

A schematic diagram illustrating the pharmacokinetics of digoxin over a 24-hour Holter time period, divided into four-hourly segments, is shown in Figure 2.

## Discussion

This prospective Holter study is, to our knowledge, the first to directly examine the temporal relationship between oral digoxin intake and intraday ventricular ectopic burden, in line with digoxin pharmacokinetics. In this cohort of patients on stable digoxin therapy for HFrEF or RHD, we found no significant increase in ectopic burden during the pharmacokinetic peak phase (hours +2 to +6 post-ingestion) compared with the trough phase or any other four-hour interval. Even the subgroup analysis of the +2 to +6 post-ingestion period, when peak digoxin effect was expected, did not reveal any increase in ectopic. This implies that the arrhythmias do not correlate with varying blood digoxin levels over the 24-hour period.

Although digoxin effectively alleviates symptoms in patients with heart failure and atrial fibrillation, its clinical application has been restricted due to worries about arrhythmias and sudden death [5,14]. No study has examined the risk of arrhythmias associated with daily variations in digoxin blood levels. This is a very pertinent question. The mechanistic rationale for our hypothesis was based on the principle that digoxin has a dose-dependent effect.

Transient supratherapeutic concentrations at peak absorption might generate a window of heightened arrhythmogenicity. Our findings argue against this for ectopic arrhythmias at the current therapeutic dosing. A possible explanation is that the clinically relevant arrhythmogenic threshold is not transiently exceeded during the normal pharmacokinetic peak phase in patients on 0.25 mg once daily with intact renal function. This would be consistent with the observation from the ARISTOTLE sub-analysis, which demonstrated that mortality risk escalated sharply only when trough serum concentrations were chronically exceeded 1.2 ng/mL [7].

These findings have clinical implications. Concerns about intra-day arrhythmic risk have occasionally been invoked to support divided dosing or modified-release formulations of digoxin. Our data do not support a pharmacokinetic rationale for this practice regarding ectopic burden. However, it must be emphasised that our study assessed only ectopic arrhythmias; no sustained ventricular tachyarrhythmias were observed, limiting generalisability to life-threatening arrhythmia endpoints. The substrate-dependent nature of digoxin toxicity, well-established in the setting of hypokalaemia, hypomagnesaemia, and renal dysfunction, likely exerts a far greater influence on arrhythmic risk than intra-day concentration fluctuations.

The mechanisms underlying life-threatening digoxin-associated arrhythmias may involve a different concentration threshold, a different time course, or a different interaction with metabolic cofactors (hypokalaemia, acidosis, or ischaemia) that were neither measured nor experimentally manipulated in this study. The dip after the +8 to +12 h timepoint (approaching late afternoon) likely reflects the circadian-driven suppression of ectopic [15]. Late afternoon may be associated with reduced sympathetic drive and circulating catecholamines, which may underlie this phenomenon.

The DIG trial’s signal of increased arrhythmic mortality in the digoxin arm is not contradicted by the current study findings [3]. Rather, it reflects chronic exposure effects, interaction with the underlying myocardial substrate, and out-of-range serum levels, rather than a pharmacokinetic peak phenomenon. Our null result in a heterogeneous but clinically representative cohort is complementary to this picture. Our findings suggest that once-daily dosing of standard 0.25 mg digoxin does not create a predictable within-day arrhythmia hazard window. However, assessment of chronic exposure effects requires larger studies in future. The proarrhythmic concern may be related to the patient-specific electrophysiological substrate rather than the digoxin effect.

Limitations

Multiple limitations must be acknowledged. Firstly, digoxin serum levels were not assessed, but the peak and trough designations are pharmacokinetic surrogates derived from published data. It may not accurately reflect true peak tissue concentrations in all patients, particularly those with variable gastrointestinal absorption or advanced heart failure. Another limitation is the interaction of concomitant medications, which were not prospectively assessed. Also, the assessment of chronic exposure effects may require larger controlled studies in the future. Other limitations are a modest sample size and a heterogeneous patient population. The circadian variation in ectopic activity might interfere with analysis, but it usually leads to false associations rather than hiding real ones [15]. Variable digoxin clearance times in HFrEF patients also reduce circadian confounding. Hence, it is likely that the absence of correlation reflects genuine variation in individual drug response rather than a circadian variation.

Future studies should incorporate concurrent serum digoxin level measurement and restrict the population to a homogeneous HFrEF cohort, adequately powered for the primary endpoint.

## Conclusions

In a prospective cohort of patients with established HFrEF or RHD receiving maintenance oral digoxin, the temporal distribution of ventricular ectopic burden does not correlate with the drug's pharmacokinetic peak or trough, as assessed by 24-hour Holter monitoring. These hypothesis-generating data suggest that intra-day concentration fluctuation at standard therapeutic doses may not be a primary driver of digoxin-related ectopic arrhythmias. Adequately powered studies with concurrent serum level monitoring are required to confirm this finding and determine its applicability to sustained ventricular arrhythmias.
